# Ethanolamine plasmalogens increase luteinising hormone pulsatility and are associated with GPR61 expression in bovine gonadotrophin‐releasing hormone neurons

**DOI:** 10.1111/jne.70273

**Published:** 2026-09-24

**Authors:** Hiroya Kadokawa, Tomoaki Kubo, Kana Kobayashi, Denis Karani Wanjiru, Yvan Bienvenu Niyonzima

**Affiliations:** ^1^ Joint Faculty of Veterinary Medicine Yamaguchi University Yamaguchi‐shi Yamaguchi‐ken Japan; ^2^ Dairy Cattle Group, Hokkaido Research Organization Dairy Research Center Nakashibetsu Japan

**Keywords:** cattle, ethanolamine plasmalogens, GnRH neurons, GPR61, LH pulsatility

## Abstract

Pulsatile secretion of luteinising hormone (LH) reflects episodic activity of hypothalamic gonadotrophin‐releasing hormone (GnRH) neurons and is essential for reproductive function. Plasmalogens are ether phospholipids enriched in neuronal membranes; however, their role in neuroendocrine regulation of reproduction remains unclear. We examined the effects of ethanolamine and choline plasmalogens on LH pulsatility in vivo and investigated GPR61 expression in bovine hypothalamic tissues and relevant cell lines. The oestrous cycles of 15 Holstein heifers were synchronised, and blood samples were collected at 10 min intervals for 8 h. Ethanolamine plasmalogens, choline plasmalogens or vehicle were administered intravenously. LH concentrations were measured by radioimmunoassay, and LH pulses were identified using the Pulsar algorithm. GPR61 expression was examined by RT‐PCR, Western blotting and immunofluorescence in bovine hypothalamic tissues and in cell lines. LH pulse frequency showed a significant treatment × time interaction (*p* < .01) and increased after ethanolamine plasmalogen administration (Holm‐adjusted *p* = .0084), whereas no significant changes were observed after choline plasmalogen or control treatments. Mean LH concentrations showed a significant effect of time (*p* < .01), whereas neither the treatment effect nor the treatment × time interaction was significant. LH pulse amplitude was unaffected. GPR61 was expressed in GnRH neurons in the bovine hypothalamus, including the preoptic area, arcuate nucleus and median eminence, and was also detected in GT1‐7 and LβT2 cells. Ethanolamine plasmalogens are associated with increased LH pulsatility in vivo and GPR61 expression in GnRH neurons, suggesting a potential role for plasmalogen composition in the neuroendocrine regulation of reproduction.

## Graphical abstract

Ethanolamine plasmalogens increased LH pulse frequency. GPR61 was expressed in GnRH neurons and gonadotrophs, suggesting a role in neuroendocrine regulation. Ethanolamine plasmalogens (EPls), but not choline plasmalogens (CPls), may act via GPR61 expressed in GnRH neurons in the preoptic area and in anterior pituitary gonadotrophs. This pathway may modulate GnRH secretion and LH release, with additional direct effects on gonadotrophs. This model is consistent with in vivo and in vitro findings.

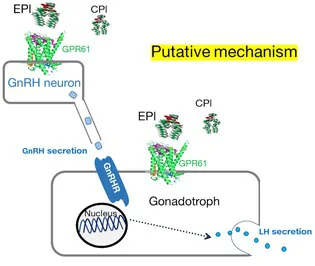

## 1 INTRODUCTION

Plasmalogens are ether phospholipids enriched in neuronal membranes and are implicated in membrane organisation and cellular signalling.^1^, ^2^ Reductions in plasmalogen levels have been reported in age‐related neurodegenerative disorders, suggesting an important role in maintaining neuronal function.^3^, ^4^ However, their roles in neuroendocrine regulation remain largely unexplored.

Plasmalogens are classified into ethanolamine plasmalogens (EPls) and choline plasmalogens (CPls). EPls are enriched in brain tissues, whereas CPls are more abundant in circulating plasma in cattle.^5^ Because of their preferential localisation in neuronal membranes, EPls may exert specific effects on neuronal signalling distinct from those of CPls.

Reproductive function in mammals is regulated by the hypothalamic–pituitary–gonadal axis. Pulsatile secretion of gonadotrophin‐releasing hormone (GnRH) drives episodic luteinising hormone (LH) release, and LH pulse frequency reflects GnRH neuronal activity.^6^, ^7^ In cattle, frequent blood sampling enables robust and reliable evaluation of LH pulsatility, providing a useful in vivo model for assessing hypothalamic–pituitary function.^8^, ^9^

Although neuropeptides regulating LH pulsatility have been extensively characterised, the role of lipid‐derived signalling remains unclear. Our previous studies demonstrated that EPls stimulate LH secretion in cultured bovine anterior pituitary cells, whereas corresponding CPl species do not.^10^, ^11^ In addition, exogenous plasmalogens can increase circulating and brain levels within hours,^12^, ^13^ suggesting that plasmalogens may influence neuroendocrine function in vivo.

One potential mechanism involves G protein‐coupled receptors. The orphan receptor GPR61 is expressed in the brain and has been implicated in neuronal signalling.^14^, ^15^ Structural analyses indicate that GPR61 couples with Gαs proteins,^16^ and our previous studies suggest that specific EPl molecular species may interact with GPR61, whereas corresponding CPl species do not.^10^, ^11^

In particular, it remains unclear whether plasmalogens differentially regulate LH pulsatility in vivo through distinct molecular mechanisms. Therefore, the objective of the present study was to determine whether administration of plasmalogens alters LH pulsatility in Holstein dairy heifers. We compared the effects of EPls and CPls and examined GPR61 expression in GnRH neurons in the bovine hypothalamus, including the preoptic area (POA), arcuate nucleus (ARC) and median eminence (ME), as well as in the mouse GnRH neuronal cell line GT1‐7 and the gonadotroph cell line LβT2.

## 2 MATERIALS AND METHODS

### 2.1 Animals and treatments for LH‐pulse measurement in vivo

All experiments were performed in accordance with the Guiding Principles for the Care and Use of Experimental Animals in the Field of Physiological Sciences (Physiological Society of Japan) and were approved by the Committee on Animal Experiments of Yamaguchi University (Approval No. 652) and the Hokkaido Research Organization (Approval No. 20250519‐09).

A total of 15 Holstein dairy heifers (9.6 ± 0.2 months old; 320 ± 8.0 kg body weight) were housed at the Animal Center of the Hokkaido Research Organization Dairy Research Center. Animals were individually housed in calf stalls and managed according to the Japanese Feeding Standard for Dairy Cattle.^17^

Oestrous cycles were synchronised using a CIDR‐Synch protocol as described previously.^18^ A CIDR device was inserted, and 1 mg estradiol benzoate was administered intramuscularly. Nine days later, 25 mg prostaglandin F₂α (Pronalgon F, Zoetis Inc., Tokyo, Japan) was administered once. Two days later, 100 μg GnRH (Spornen, Kyoritsu Seiyaku Corporation, Tokyo, Japan) was administered intramuscularly to induce ovulation. Heifers were used 3–5 days after ovulation, a stage characterised by frequent LH pulsatility.^9^

Animals were randomly assigned to one of three treatments: ethanolamine plasmalogens (EPl; C18(Plasm)‐18:1 PE; CAS 144371‐68‐5; MW 730.05; Sigma‐Aldrich, USA), choline plasmalogens (CPl; C18(Plasm)‐18:1 PC; CAS 799268‐63‐6; MW 772.13; Sigma‐Aldrich, USA), or vehicle control. Each compound was mixed with 50 mL of heparinised autologous plasma collected from the same animal and infused via a jugular vein catheter over approximately 30 s.

The EPl compound was supplied as a dry powder and dissolved in ethanol (5 mg/600 μL); each animal received 1 mg in 120 μL ethanol. The CPl compound was supplied as a chloroform solution; chloroform was evaporated under nitrogen gas, and the lipid was redissolved in ethanol (5 mg/600 μL); each animal received 1 mg in 120 μL ethanol. Control animals received 120 μL ethanol mixed with 50 mL autologous plasma and administered in the same manner as the plasmalogen treatments.

A jugular vein catheter (16G; Medikit Co., Ltd., Tokyo, Japan) was inserted 1 day before the experiment to allow frequent blood sampling and was maintained by regular flushing with heparinised saline (10 U/mL). Blood samples were collected at 10 min intervals for 8 h (10:00–18:00; time −240 to 240 min). Approximately 2 mL of blood was collected at each time point, yielding approximately 1.5 mL of plasma. The total blood volume collected per animal (approximately 100 mL) was within acceptable limits for repeated sampling and did not adversely affect animal condition.

### 2.2 LH assay and LH pulse analysis

LH concentrations in plasma were assayed in duplicate by double‐antibody radioimmunoassay using ^125^I‐labelled bovine LH and anti‐ovine LH antiserum (AFP11743B and AFP192279; National Hormone and Pituitary Program, NIDDK, USA). The limit of detection was 0.40 ng/mL, and intra‐ and inter‐assay coefficients of variation were 3.9% and 6.9%, respectively, at 0.51 ng/mL.

LH pulse frequency (number of pulses per 4 h), amplitude and mean LH concentrations were determined using the Pulsar algorithm^19^ implemented in the Munro pulse analysis program (Zaristow Software, West Morham, Haddington, East Lothian, UK). LH pulses were identified automatically using the Pulsar algorithm, and identical parameter settings were applied to all LH profiles. G parameters were set at 3.0, 2.6, 1.25, 1.0 and 0.6 for G1–G5, respectively, corresponding to pulses composed of one to five consecutive samples exceeding the baseline. Baxter parameters were set at 0.1261 (b1, intercept), 0.0355 (b2, linear coefficient) and 0.0123 (b3, quadratic coefficient).

### 2.3 Antibodies used for immunohistochemistry and immunocytochemistry

Human GPR61 (451 amino acids; National Center for Biotechnology Information [NCBI] accession number AAH67464.1) shares 96.0% sequence homology with the bovine protein (449 amino acids; NCBI accession number NP_001101185.2) and 95.8% with the mouse protein (449 amino acids; NCBI accession number NP_001292390.1). A rabbit polyclonal anti‐GPR61 antibody directed against the extracellular region of human GPR61 (ORB183901; Biorbyt, Cambridge, UK) was used. The peptide antigen corresponds to amino acids 4–18 (SPIPQSSGNSSTLGR) within the N‐terminal extracellular domain. This sequence is 100% conserved in bovine and mouse GPR61 and shows no significant homology to other proteins in these species based on BLAST analysis using sequences retrieved from DNA Data Bank of Japan, GenBank and European Bioinformatics Institute databases. The specificity of this antibody has been previously validated in bovine tissues by western blotting and immunohistochemistry.^14^ Given the complete conservation of the antigen epitope, this antibody is expected to recognise GPR61 consistently in bovine tissues, including brain regions.

An anti‐GnRH mouse monoclonal antibody (clone GnRH I HU11B: sc‐32292; Santa Cruz Biotechnology, Dallas, TX), raised against a synthetic GnRH I decapeptide,^20^ was also used. This antibody has been widely validated for the detection of GnRH neurons in the mammalian brain, including bovine tissue, and was used for immunohistochemistry to visualise GnRH neurons.^21^

### 2.4 Animals and treatments for RT‐PCR, western blots and immunofluorescence staining

Brain samples were collected from Japanese Black cattle managed by a contracted farmer in western Japan, where the first author's institution is located. Japanese Black heifers were used because they are the predominant breed of heifers available from local slaughterhouses in this region, whereas Holstein heifers are rarely available for tissue collection. The cattle were fed twice daily with a total mixed ration according to the Japanese Feeding Standard for beef Cattle.^22^ All cattle were traceable through Japan's national livestock registration system, ensuring verified breed and age information. Animals were slaughtered at a local abattoir in accordance with regulations of the Ministry of Agriculture, Forestry and Fisheries of Japan. All animals were non‐lactating, non‐pregnant and free from ovarian abnormalities, such as follicular or luteal cysts.^23^

Brain samples consisting of an anterior hypothalamic block containing the preoptic area (POA) and a posterior hypothalamic block containing the arcuate nucleus (ARC) and median eminence (ME) were obtained from healthy post‐pubertal Japanese Black heifers (22.5 ± 1.6 months of age; *n* = 5; young group) using methods described previously.^21^, ^24^, ^25^, ^26^ Each block was fixed in 4% paraformaldehyde at 4°C for 24 h, cryoprotected in 20–30% sucrose and stored until sectioning.

Additional anterior and posterior hypothalamic tissue samples were collected from healthy post‐pubertal Japanese Black heifers (22.5 ± 1.4 months of age; *n* = 5) for reverse transcription–polymerase chain reaction (RT‐PCR) and Western blotting, using methods described previously.^25^, ^26^ The anterior and posterior hypothalamic regions were defined according to a bovine brain atlas,^27^, ^28^ dissected bilaterally, snap‐frozen in liquid nitrogen and stored at −80°C until RNA or protein extraction.

### 2.5 Cell culture for GT1‐7 and LβT2 cells

The GT1‐7 hypothalamic neuronal cells and LβT2 gonadotroph cells were generous gifts from Dr. Pamela Mellon.^29^, ^30^ Cells were maintained in Dulbecco's Modified Eagle's Medium (DMEM; Thermo Fisher Scientific, Waltham, MA) supplemented with 10% foetal bovine serum, 100 U/mL penicillin and 50 μg/mL streptomycin, and cultured at 37°C in a humidified atmosphere with 5% CO_2_. Cells were used for RT‐PCR and immunocytochemistry.

### 2.6 RT‐PCR and sequence verification

RT‐PCR was performed as described previously for bovine hypothalamic tissues and GT1‐7 and LβT2 cells.^26^, ^31^ Total RNA was extracted using RNAzol RT (Molecular Research Center, Cincinnati, OH). RNA quality was verified by electrophoresis with ethidium bromide staining, and 28S:18S ratios were approximately 2:1. Total RNA was treated with DNase prior to reverse transcription to eliminate potential genomic DNA contamination, and complementary DNA (cDNA) was synthesised using the Verso cDNA Synthesis Kit (Thermo Fisher Scientific).

PCR was conducted using primers designed from bovine GPR61 (NCBI reference sequence NM_001038571.2) and mouse Gpr61 (NM_001305461.2 and NM_001411298.1). Primer sequences are shown in Table 1. Primers were designed using Primer Express software (version 3.0.1; Applied Biosystems, Foster City, CA), and specificity was confirmed by BLAST. Primers were designed in regions common to the transcript variants, allowing amplification of both mouse *Gpr61* variants. Due to sequence constraints and exon structure of the transcripts, primer design across exon boundaries was not feasible; therefore, primers were designed within a single exon. PCR was performed using a Veriti 96‐Well Thermal Cycler (Thermo Fisher Scientific) with 50 ng of cDNA and Tks Gflex DNA Polymerase (Takara Bio Inc., Shiga, Japan). The thermocycling conditions were as follows: initial denaturation at 94°C for 1 min, followed by 35 cycles of 98°C for 10 s, 60°C for 15 s and 68°C for 30 s. PCR products were separated on a 1.5% agarose gel and visualised using GelStar nucleic acid gel stain (Lonza, Basel, Switzerland). PCR products were purified using the NucleoSpin Extract II kit (Takara Bio Inc.) and sequenced using an ABI 3130 Genetic Analyzer (Thermo Fisher Scientific) with one of the PCR primers and the BigDye Terminator v3.1 Cycle Sequencing Kit (Thermo Fisher Scientific). The sequences were analysed by BLAST against the NCBI database.

**TABLE 1:** Details of primers for bovine or mouse genes used for reverse transcription–polymerase chain reaction.

Gene, species	Primer sequence 5′–3′		Position		Size (bp)
			Nucleotide	Exon	
*GPR61*, bovine	F	CCCTAGGCTGCTCACTCCAA	1300–1319	3	473
	R	CCGGATCTGCCGATTAAGAC	1753–1772	3	
*GPR61*, mouse	F	TGGCCTCCCCGATCTATCTC	2279–2298	2	290
	R	TCCGCTGAAAATACCATTGCT	2548–2568	2	

### 2.7 Western blotting

Western blotting was performed as described previously.^25^, ^26^ Total protein was extracted using lysis buffer containing protease inhibitors. Proteins were separated by SDS–PAGE and transferred to PVDF membranes. Total protein was visualised using Revert 700 stain (LI‐COR Biosciences, Lincoln, NE), and membranes were scanned using an Odyssey CLx system (LI‐COR Biosciences).

Membranes were blocked using Can Get Signal Immunoreaction Enhancer (Toyobo, Osaka, Japan) and incubated with anti‐GPR61 antibody (1:100,000) at 4°C for 16 h, followed by horseradish peroxidase‐conjugated goat anti‐rabbit IgG (Bethyl Laboratories, Montgomery, TX; 1:100,000) for 1 h at room temperature. Protein bands were detected using ECL Prime (Cytiva, Marlborough, MA) and imaged with an ImageQuant 800 system (Cytiva).

Signal specificity was confirmed by omission of the primary antibody, and by substitution with normal rabbit IgG. GPR61 signal intensity was normalised to total protein content.

### 2.8 Tissue preparation and immunofluorescence staining

Hypothalamic blocks were embedded in OCT compound and frozen. Serial coronal sections (50 μm thickness) were cut using a cryostat (CM1900; Leica Microsystems, Wetzlar, Germany) as described previously,^24^ with reference to a bovine brain atlas.^27^, ^28^ The anterior hypothalamic block included the POA, and the posterior block included the ARC and ME. The POA was identified as the hypothalamic region dorsal to the optic chiasm and adjacent to the third ventricle. The ARC was identified at the level of the characteristic funnel‐shaped infundibular recess of the third ventricle adjacent to the ME.^28^

Sections were stored at −30°C in PBS containing 50% glycerol, 250 mM sucrose and 3.2 mM MgCl_2_·6H_2_O as described previously,^32^ until immunostaining. Free‐floating sections were thawed, washed in PBS and permeabilised with PBS containing 0.5% Tween 20 for 3 min. Autofluorescence was reduced using a combination of glycine/hydrogen peroxide treatment^21^, ^24^ and a commercial autofluorescence quenching kit (Vector TrueVIEW; Vector Laboratories, Burlingame, CA).

Sections were blocked in PBS containing 2% normal goat serum, 50 mM glycine, 0.05% Tween 20, 0.1% Triton X‐100 and 0.1% BSA for 30 min, followed by incubation at 4°C for 16 h with primary antibodies (anti‐GnRH mouse and anti‐GPR61 rabbit; both diluted 1:1000). After washing, sections were incubated for 4 h at room temperature with Alexa Fluor 488‐labelled goat anti‐rabbit IgG (A‐11034; Thermo Fisher Scientific, Waltham, MA) and Alexa Fluor 546‐labelled goat anti‐mouse IgG (A‐11030; Thermo Fisher Scientific) (1 μg/mL), together with DAPI (1 μg/mL; FUJIFILM Wako Pure Chemicals, Osaka, Japan). Sections were washed and treated again with the autofluorescence quenching reagent, mounted on glass slides (MAS‐coated Superfrost; Matsunami Glass, Osaka, Japan) and coverslipped using ProLong Glass Antifade Mountant (Thermo Fisher Scientific).

Fluorescence images were acquired using a confocal microscope (LSM710; Carl Zeiss, Göttingen, Germany) equipped with 405, 488, 533 and 633 nm lasers. Images were obtained using 20× or 40× objectives and analysed using ZEN 2012 Black Edition software (Carl Zeiss). GnRH and GPR61 localisation was examined in double‐labelled sections.

Control sections were processed either without primary antibodies or incubated with normal rabbit IgG and normal mouse IgG (both FUJIFILM Wako Pure Chemicals) at the same concentration as the respective primary antibodies. No immunoreactive signals were detected in control sections.

Neuronal structures were defined as follows: cell bodies were identified as round or polygonal structures with a diameter >8 μm, and fibres as continuous or punctate immunopositive signals. GnRH neurons were identified based on morphology and GnRH immunoreactivity, as described previously.^21^

Co‐localisation was assessed by overlapping GnRH (red) and GPR61 (green) signals, appearing as yellow in merged images. Analyses were performed in single optical sections to avoid artefacts from different focal planes. For each animal, at least four sections containing the POA and four sections containing the ARC and ME were analysed.

### 2.9 Immunocytochemistry and microscopy

LβT2 cells (1.0 × 10^4^ cells per well) were cultured in μ‐Slide 8 Well chambers (ibidi GmbH, Martinsried, Germany) pre‐coated with poly‐D‐lysine, whereas GT1‐7 cells were seeded onto uncoated chambers. Cells were cultured for 3 days under standard conditions.

Cells were fixed with 4% paraformaldehyde for 10 min and washed with PBS. To assess GPR61 localisation at the cell surface, cells were incubated with anti‐GPR61 antibody without permeabilisation. After blocking with 1% BSA, cells were incubated with primary antibody for 4 h at room temperature, followed by Alexa Fluor 488 goat anti‐rabbit IgG (1 μg/mL) and DAPI for 2 h.

Fluorescence images were acquired using a Leica MICA imaging system with a 63× objective. Acquisition settings were kept constant within each experiment. Transmitted‐light images were obtained using integrated modulation contrast (IMC) mode. Negative controls included omission of the primary antibody and incubation with normal rabbit IgG at the same concentration as the primary antibody.

### 2.10 Statistical analysis

Statistical analyses were performed using StatView version 5.0 (SAS Institute, Cary, NC). Outliers were assessed using Grubbs' test. Normality was evaluated using Shapiro–Wilk and Kolmogorov–Smirnov tests, and homogeneity of variance was assessed using the *F* test. LH pulse parameters were analysed using two‐way repeated‐measures ANOVA (treatment × time), with time (Pre vs. Post) as a within‐subject factor. When a significant treatment × time interaction was detected, post hoc comparisons between Pre and Post within each treatment group were performed using paired *t* tests with Holm correction for multiple comparisons. To compare treatment‐induced changes among treatment groups, the Post/Pre ratio of LH pulse frequency was additionally analysed using the Kruskal–Wallis test with tie correction. Statistical significance was set at *p* < .05. Data are presented as mean ± SEM.

## 3 RESULTS

### 3.1 Effects of plasmalogens on LH pulsatility in Holstein heifers

**FIGURE 1:**
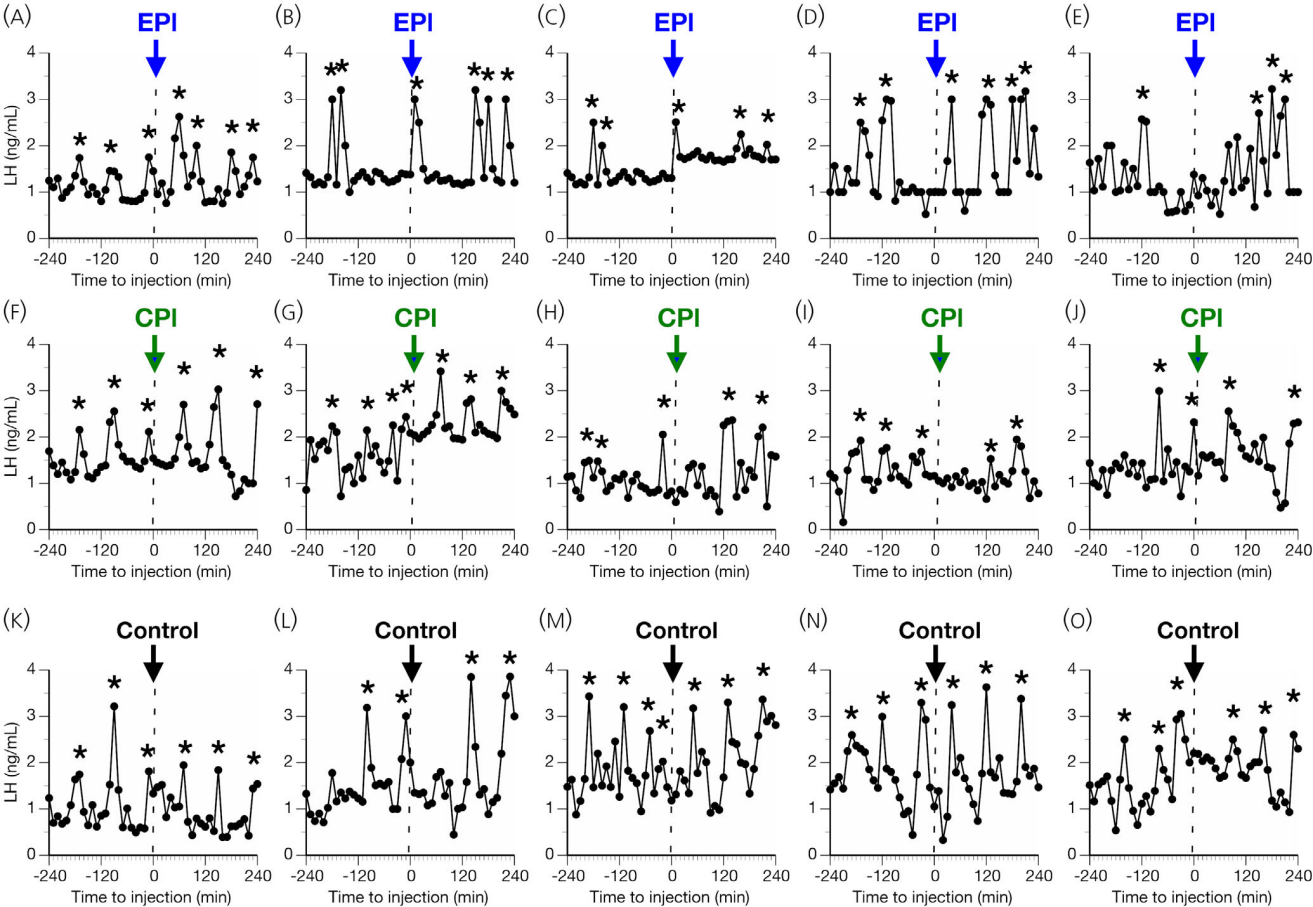
LH pulse profiles of all 15 heifers before and after treatment. Plasma LH concentrations were measured at 10 min intervals for 8 h in each animal. Treatment was administered at time 0 (arrows). LH pulses were identified using the Pulsar algorithm^19^ implemented in the Munro pulse analysis program (Zaristow Software, UK). Identical parameter settings were applied to all profiles (G1–G5: 3.0, 2.6, 1.25, 1.0 and 0.6; Baxter parameters: b1 = 0.1261, b2 = 0.0355, b3 = 0.0123). Detected pulses were automatically identified and visually inspected. Panels show all individual animals from the EPl‐treated group (A–E; *n* = 5), CPl‐treated group (F–J; *n* = 5) and control group (K–O; *n* = 5).

**FIGURE 2:**
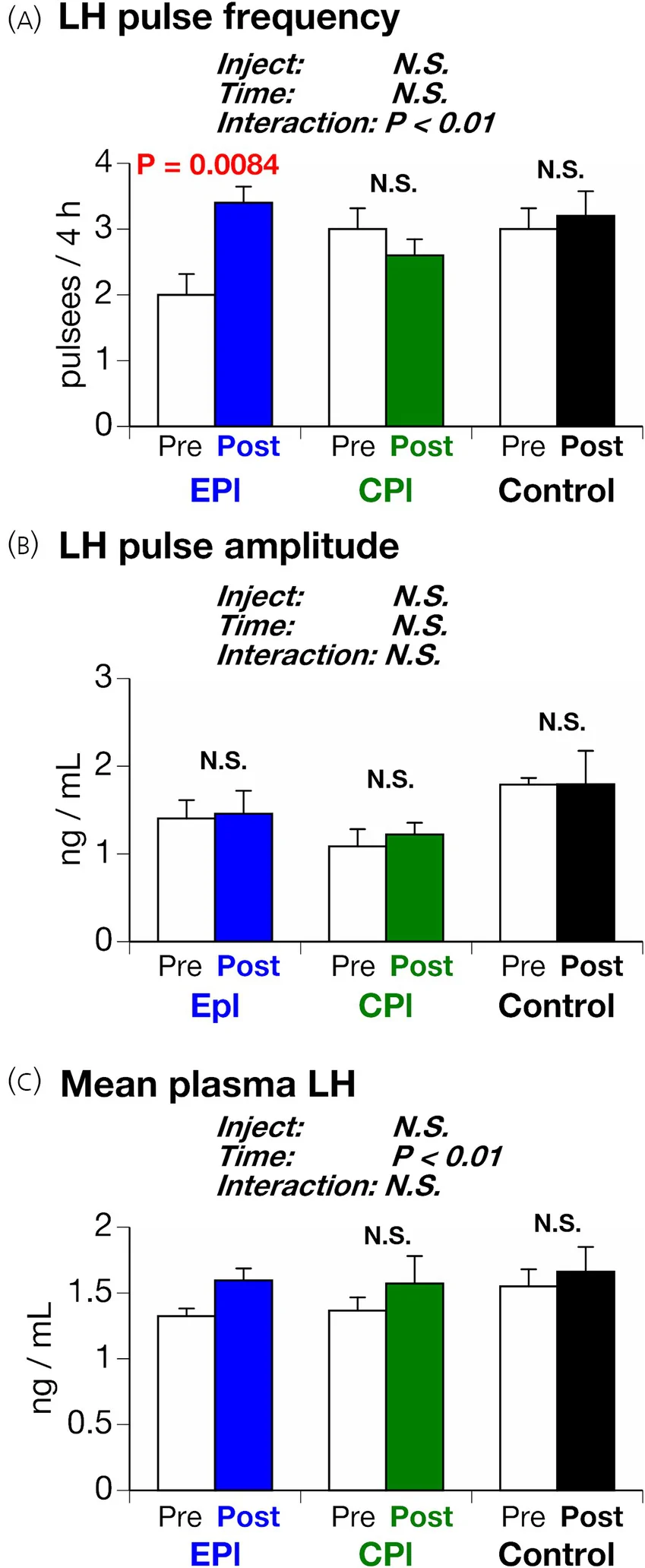
Summary analysis of LH pulsatile secretion. (A) LH pulse frequency, (B) LH pulse amplitude and (C) mean plasma LH concentration during the 4 h periods before (Pre) and after (Post) treatment. Blood samples were collected at 10 min intervals, and treatment was administered at time 0. Values are presented as mean ± SEM (*n* = 5 per group). LH pulses were detected using the Pulsar algorithm^19^ implemented in the Munro pulse analysis program, as described in the Methods section. Pulse frequency represents the number of pulses per 4 h. Pulse amplitude represents the difference between the pulse peak and the preceding nadir. Mean plasma LH represents the average concentration across each 4 h period. Data were analysed using two‐way repeated‐measures ANOVA (treatment × time), with time (Pre vs. Post) as a within‐subject factor. When a significant treatment × time interaction was detected, post hoc comparisons between Pre and Post within each treatment group were performed using paired *t* tests with Holm correction. Significant differences are indicated in the figure.

Frequent blood sampling at 10 min intervals revealed clear episodic LH secretion in Holstein heifers during the early luteal phase (Figures 1 and 2). Two‐way repeated‐measures ANOVA revealed a significant treatment × time interaction for LH pulse frequency (*F*_(2, 12)_ = 15.846, *p* = .0004), whereas neither the main effect of treatment (*F*_(2, 12)_ = 0.034, *p* = .967) nor the main effect of time (*F*_(1, 12)_ = 2.462, *p* = .143) was significant. Post hoc analysis revealed that LH pulse frequency increased after EPl administration (Holm‐adjusted *p* = .0084), whereas no significant changes were observed after CPl or control treatments (Figures 1 and 2). In addition, the Post/Pre ratio of LH pulse frequency differed significantly among treatment groups (Kruskal–Wallis test with tie correction: *H*(2) = 9.45, *p* = .0089).

Two‐way repeated‐measures ANOVA showed no significant treatment × time interaction for LH pulse amplitude (*F*_(2, 12)_ = 0.065, *p* = .937), and no significant main effect of time (*F*_(1, 12)_ = 0.184, *p* = .675) or treatment (*F*_(2, 12)_ = 2.820, *p* = .099).

Two‐way repeated‐measures ANOVA showed a significant main effect of time on mean plasma LH concentrations (*F*_(1, 12)_ = 12.002, *p* = .0047), whereas neither the treatment × time interaction (*F*_(2, 12)_ = 0.669, *p* = .531) nor the main effect of treatment (*F*_(2, 12)_ = 0.389, *p* = .686) was significant.

These results indicate that EPl, but not CPl, is associated with increased LH pulsatility.

### 3.2 Expression of GPR61 in the bovine hypothalamus and GT1‐7 and LβT2 cells

**FIGURE 3:**
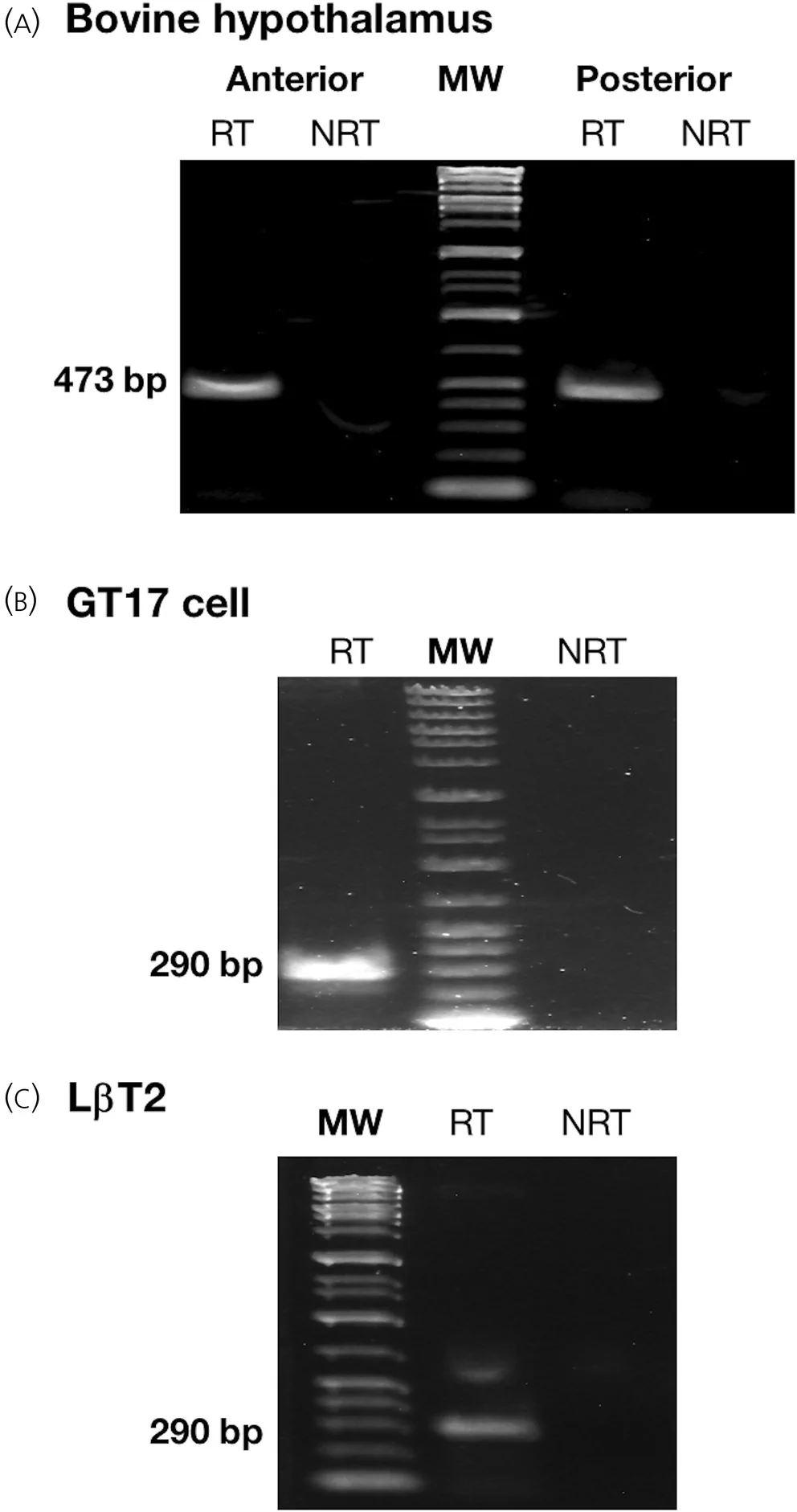
RT‐PCR analysis of *GPR61* in bovine anterior and posterior hypothalamus (A), GT1‐7 (B) and LβT2 cells (C). RNA samples were subjected to reverse transcription (RT) or processed without reverse transcription (NRT) as a negative control. Amplification products were separated by agarose gel electrophoresis and visualised with an intercalating dye. A distinct band of the expected size (473 bp for bovine *GPR61*, and 290 bp for mouse *Gpr61*) was detected in RT samples, whereas no amplification was observed in NRT controls [MW, DNA marker (100 bp ladder)].

To explore a potential mechanism underlying the effects of plasmalogens on LH pulsatility, we examined whether GPR61 is expressed in the bovine hypothalamus and GT1‐7 and LβT2 cells. RT‐PCR detected GPR61 mRNA expression in the bovine hypothalamus, and GT1‐7 and LβT2 cells (Figure 3). A single band of the expected size was observed by agarose gel electrophoresis. No amplification was detected in the corresponding no‐RT controls.

Sequence analysis of the amplified products showed the highest similarity to bovine GPR61 (NM_001038571.2) and mouse Gpr61 (NM_001305461.2), with 100% query coverage, an *E*‐value of 0.0 and 99% identity. No other bovine or mouse genes showed significant homology, confirming that the amplified products corresponded to GPR61.

### 3.3 Expression of GPR61 in GnRH neurons of the bovine hypothalamus and GT1‐7 and LβT2 cells

**FIGURE 4:**
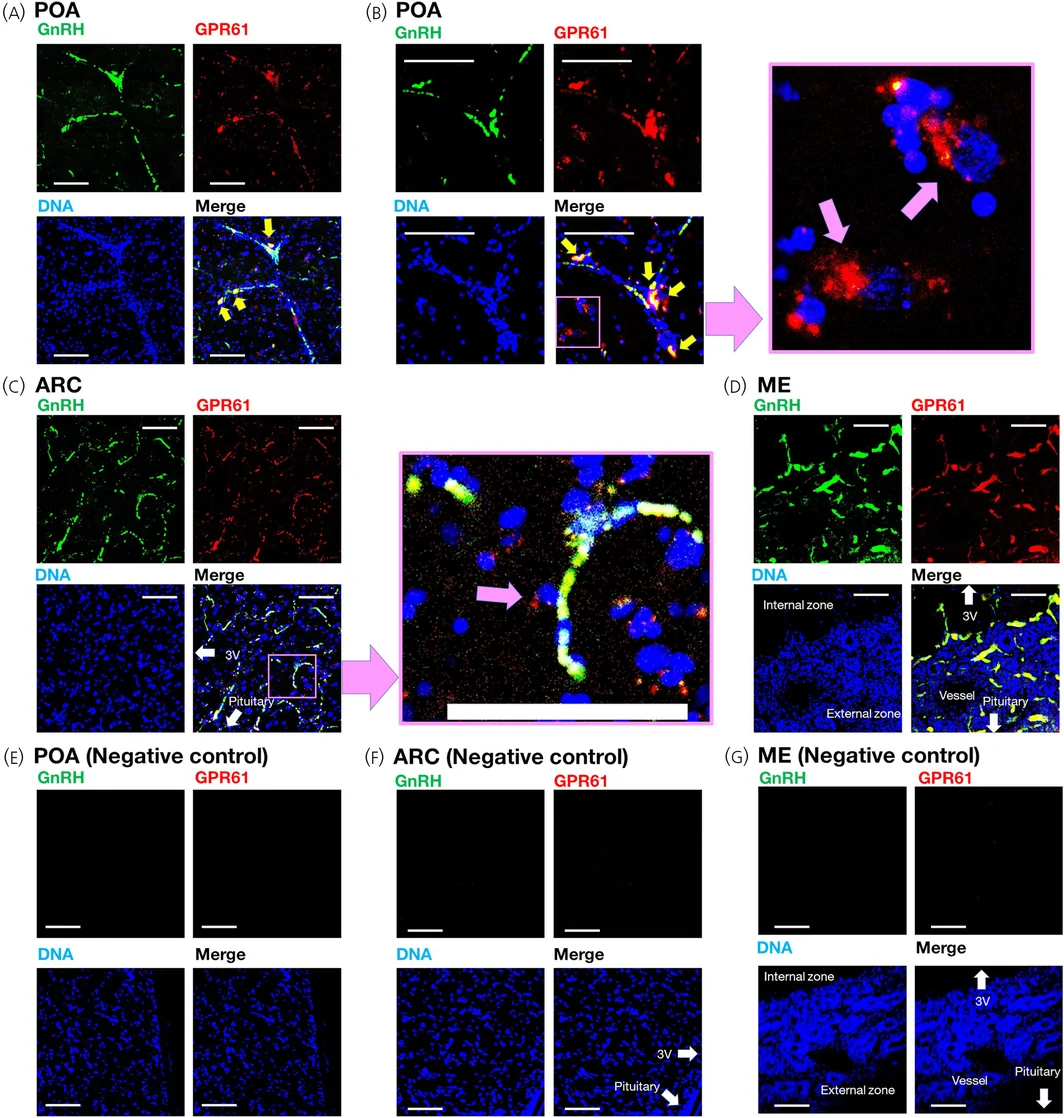
Co‐localisation of GPR61 with GnRH neurons in the bovine hypothalamus. (A) Representative immunofluorescence images from multiple animals and sections of the preoptic area (POA) showing GnRH neurons (green), GPR61 immunoreactivity (red) and nuclei (blue), together with merged images. Merged images demonstrate co‐localisation of GPR61 in GnRH neurons. (B) Representative immunofluorescence images of the POA. Yellow arrows indicate GnRH neuronal cell bodies. The boxed region in the merged image is shown at higher magnification on the right. (C) Representative images of the arcuate nucleus (ARC) showing GPR61 immunoreactivity. The boxed region in the merged image is shown at higher magnification on the right. (D) Representative image of the median eminence (ME). GPR61 immunoreactivity was observed in fibre‐like structures in the internal zone, where DAPI staining was sparse due to the low density of neuronal cell bodies. Co‐localisation was particularly evident in the external zone of the ME. (E–G) Negative control sections of the POA (E), ARC (F) and ME (G) incubated with normal rabbit and mouse IgG at the same concentration as the primary antibodies showed no immunoreactivity. Pink arrows indicate representative GPR61‐immunoreactive signals outside GnRH‐immunoreactive neurons. Scale bar = 100 μm.

GPR61 immunoreactivity was detected in GnRH neurons in the bovine POA (Figure 4A,B). Confocal microscopy demonstrated co‐localisation of GPR61 (red) with GnRH‐immunoreactive neuronal cell bodies (green). Overall, 164 of 168 GnRH neurons (97.6%) were immunopositive for GPR61. GnRH‐immunoreactive fibres were also observed in the ARC (Figure 4C) and ME (Figure 4D), where GPR61 immunoreactivity was detected along these fibres, including in the external zone of the ME. In addition to GnRH neurons, representative GPR61‐immunoreactive signals outside GnRH‐immunoreactive neurons were also observed in the POA and ARC (Figure 4A,C, pink arrows). These signals were not quantified, and their cellular identity was not determined in the present study. No immunoreactivity was observed in control sections incubated with normal rabbit and mouse IgG at the same concentration as the primary antibodies in the POA (Figure 4E), ARC (Figure 4F) and ME (Figure 4G), or in sections processed without primary antibodies (data not shown).

**FIGURE 5:**
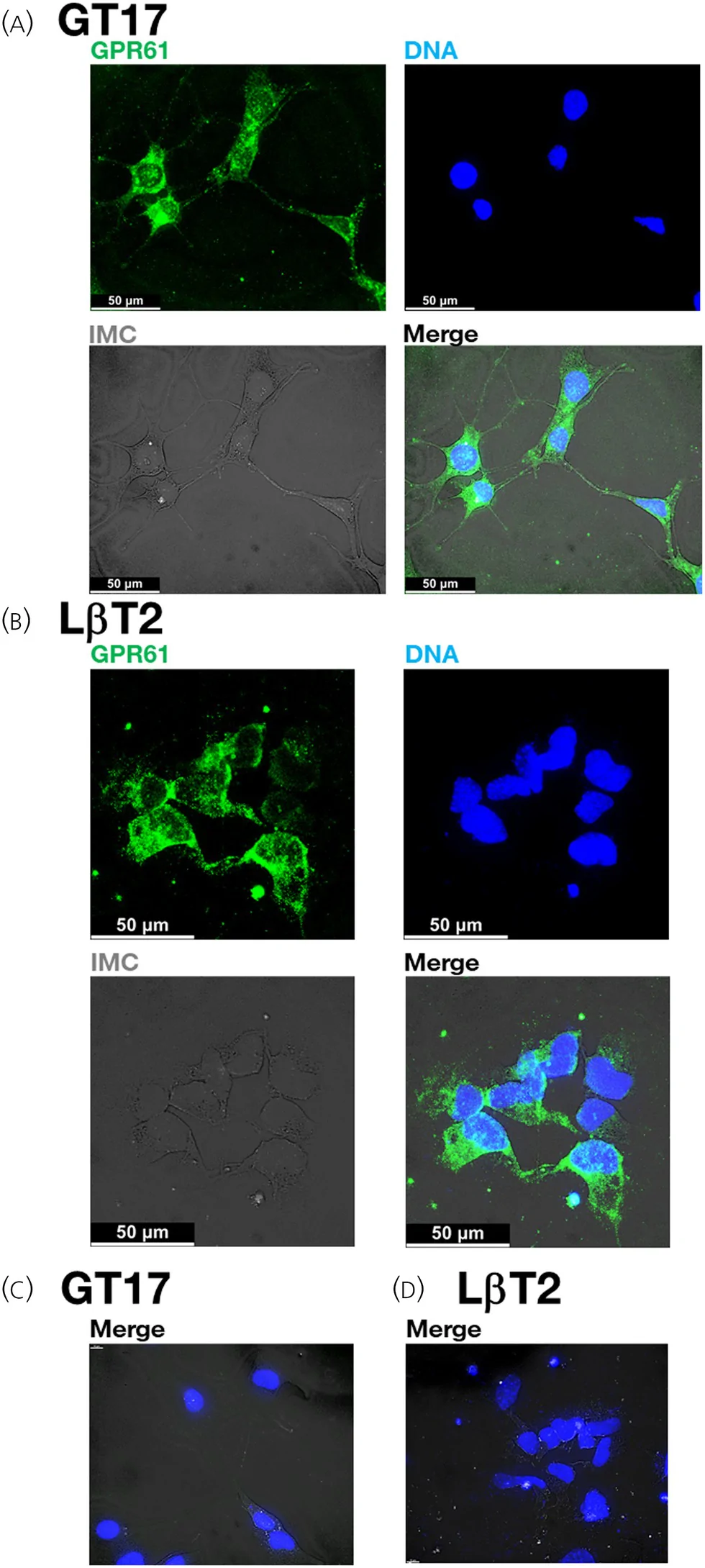
Expression of GPR61 in GT1‐7 and LβT2 cells under non‐permeabilised conditions. (A) GT1‐7 cells and (B) LβT2 cells stained for GPR61 (green) and nuclei (DAPI, blue), together with IMC and merged images. Merged images indicate predominant localisation of GPR61 at the cell periphery. (C) Merged image of control GT1‐7 cells and (D) merged image of control LβT2 cells incubated with normal rabbit IgG at the same concentration as the primary antibody. Scale bars = 50 μm.

Immunofluorescence staining demonstrated GPR61 protein expression in GT1‐7 (Figure 5A) and LβT2 cells (Figure 5B). No immunoreactivity was observed in control cells incubated with normal rabbit IgG at the same concentration as the primary antibody in GT1‐7 (Figure 5C) and LβT2 cells (Figure 5D), or in cells processed without primary antibody (data not shown).

## 4 DISCUSSION

GnRH neurons play a central role in the regulation of reproductive function, and pulsatile GnRH secretion is the primary driver of LH release from anterior pituitary gonadotrophs.^6^, ^7^ The importance of GnRH as a key regulator of reproductive endocrine function has been well established. Among the regulatory mechanisms controlling GnRH neuronal activity, KNDy (kisspeptin/neurokinin B/dynorphin) neurons are recognised as a major component of the GnRH pulse generator.^33^, ^34^, ^35^ These neurons integrate steroidal and metabolic signals and play a crucial role in generating pulsatile GnRH secretion. However, GnRH neurons and gonadotrophs express multiple receptors and are subject to diverse regulatory mechanisms beyond classical neuropeptide signalling. For example, receptor organisation within membrane microdomains such as lipid rafts has been shown to influence signalling processes.^36^, ^37^ In addition, orphan G protein‐coupled receptors, including GPR61, which has been proposed as a regulator of gonadotroph function,^14^, ^38^ may also contribute to this regulation. These observations suggest that membrane lipid composition may represent an additional layer of regulation within the hypothalamic–pituitary axis.

The significant treatment × time interaction further supports the selective effect of EPls on LH pulsatility. The present study demonstrates that EPls increase LH pulsatility in vivo in Holstein heifers. EPl increased LH pulse frequency without affecting pulse amplitude. Mean LH concentrations showed an overall effect of time, but no treatment effect or treatment × time interaction was detected. Because LH pulse frequency is widely used as an indirect index of GnRH neuronal activity, these findings suggest that EPls may influence hypothalamic regulation of GnRH secretion.

The selective increase in LH pulse frequency without a change in amplitude suggests that EPls primarily affect hypothalamic GnRH neuronal activity rather than pituitary responsiveness. This interpretation is consistent with the detection of GPR61 in GnRH neurons in the POA in the present study. In addition, GPR61 immunoreactivity was observed in the ARC and ME, indicating that plasmalogen‐related signalling may occur at multiple hypothalamic sites.

Previous studies using cultured bovine anterior pituitary cells have demonstrated that ethanolamine plasmalogens stimulate basal and GnRH‐induced LH secretion, whereas corresponding choline plasmalogens show limited or no effect.^10^, ^11^ Furthermore, GPR61 has been reported to colocalise with GnRH receptors in lipid raft microdomains of gonadotrophs.^14^, ^38^ Together, these findings support the possibility that plasmalogen‐related signalling may interact with GnRH receptor‐associated pathways.

The marked difference between EPl and CPl observed in the present study highlights the importance of molecular structure. Although both subclasses share a common ether lipid backbone, they differ in their polar head groups and fatty acid composition. Previous studies have suggested that specific EPl molecular species may interact with GPR61, whereas corresponding CPl species do not.^10^, ^11^ These observations indicate that structural features of plasmalogens may be critical for their biological activity.

Membrane microdomains such as lipid rafts are known to organise receptor signalling.^37^ GnRH receptors have been reported to localise within these domains in gonadotrophs,^36^ and GPR61 has also been associated with similar membrane environments.^14^, ^38^ The rapid increase in LH pulse frequency observed after intravenous administration is consistent with a receptor‐mediated mechanism. Although our data do not distinguish between GPR61‐mediated signalling and indirect effects mediated through changes in membrane properties, the rapid onset of the response may favour a receptor‐mediated mechanism. These mechanisms are not mutually exclusive and may act in concert. Therefore, EPls may influence GnRH neuronal activity, potentially through interactions with GPR61‐mediated signalling and/or by modifying the membrane microenvironment. KNDy neurons are widely recognised as key regulators of the GnRH pulse generator. Although the present study demonstrated GPR61 expression in GnRH neurons, whether GPR61 is also expressed in KNDy neurons remains unknown. Therefore, EPls may regulate GnRH pulse frequency by acting directly on GnRH neurons and/or indirectly through the KNDy neuronal network. Further studies are required to distinguish these possibilities.

Accumulating evidence indicates that plasmalogens are involved in age‐related changes in brain function. EPls decrease in the bovine brain with age, and this reduction has been associated with altered gonadotroph function.^39^ In addition, circulating plasmalogen concentrations changed dynamically around the first postpartum ovulation and were associated with production traits (milk yield and milk fat percentage) and reproductive endocrine parameters (circulating concentrations of anti‐Müllerian hormone and follicle‐stimulating hormone) in dairy cattle.^5^ These findings raise the possibility that age‐related alterations in plasmalogen composition may influence hypothalamic regulation of GnRH secretion and contribute to reproductive decline.

The present findings should also be interpreted in the context of tissue distribution. EPls are enriched in brain tissues, whereas CPls are more abundant in circulating plasma.^5^, ^11^ These differences may partly explain why EPls, but not CPls, affected LH pulsatility in vivo. In addition, exogenously administered plasmalogens have been reported to increase their levels in blood and brain,^13^ supporting the physiological relevance of the present in vivo approach.

Several limitations should be considered. The present study examined a single molecular species of plasmalogens, and the effects of other structurally distinct species remain to be determined. Although GPR61 expression was demonstrated in GnRH neurons, functional validation of receptor‐mediated mechanisms in vivo was not performed. Representative GPR61‐immunoreactive signals outside GnRH‐immunoreactive neurons were also observed in the POA and ARC (Figure 4A,C, pink arrows); however, these signals were not quantified, and their cellular identity was not determined because the primary objective of the present study was to determine whether GnRH neurons express GPR61 rather than to characterise other GPR61‐positive cell populations. The number of animals used was also limited because of the technical demands of frequent blood sampling in cattle. The detection of GPR61/Gpr61 transcripts in hypothalamic tissue should not be taken as evidence of expression in GnRH neuronal cell bodies. Finally, because the present study was conducted exclusively during the early luteal phase, it remains to be determined whether the effects of ethanolamine plasmalogens vary across different stages of the oestrous cycle and whether they are influenced by progesterone‐dependent negative feedback.

In conclusion, the present study suggests that a defined molecular species of ethanolamine plasmalogen is associated with the regulation of LH pulsatility in vivo and that GPR61 is expressed in GnRH neurons in the bovine hypothalamus. These findings suggest that membrane lipid composition may contribute to the regulation of the hypothalamic–pituitary axis, in addition to established neuropeptide‐based mechanisms.

## AUTHOR CONTRIBUTIONS

**Hiroya Kadokawa:** Conceptualization; investigation; funding acquisition; writing – original draft; methodology; validation; visualization; writing – review and editing; formal analysis; project administration; data curation; supervision; resources. **Yvan Bienvenu Niyonzima:** Investigation; writing – original draft; methodology; writing – review and editing; data curation. **Tomoaki Kubo:** Conceptualization; investigation; writing – original draft; methodology; validation; data curation; resources; writing – review and editing; formal analysis. **Denis Karani Wanjiru:** Investigation; methodology; writing – original draft; writing – review and editing; data curation. **Kana Kobayashi:** Investigation; conceptualization; methodology; validation; data curation; writing – review and editing.

## FUNDING INFORMATION

This research was partly supported by a Grant‐in‐Aid for Scientific Research [JSPS KAKENHI grant numbers 24K01910 and 25K22421] from the Japan Society for the Promotion of Science (Tokyo, Japan) to Hiroya Kadokawa. The funder had no role in study design, data collection and analysis, the decision to publish, or preparation of the manuscript.

## CONFLICT OF INTEREST STATEMENT

The authors declare no conflicts of interest.

## ETHICS STATEMENT

All procedures involving animals were conducted in accordance with the guidelines of the relevant institutional and national committees on animal care and use. The experimental protocols were approved by the Committee on Animal Experiments of Yamaguchi University and the Hokkaido Research Organization.

## Data Availability

The data that support the findings of this study are available from the corresponding author upon reasonable request.
